# Supplementary material for: Mechanisms governing the pioneering and redistribution capabilities of the non-classical pioneer PU.1
Source: Nat Commun. 2020 Jan 21;11:402. doi: 10.1038/s41467-019-13960-2 (PMC6972792; doi:10.1038/s41467-019-13960-2)
Supplement: Supplementary file 7 — Source data [file 41467_2019_13960_MOESM7_ESM.zip › Source_Data/FigureS2/FigureS2B_MotifScanOutput/KmeansCluster3/homerResults/motif20.similar.html]

motif20

## Information for motif20

C
A
T
G
A
C
G
T
A
C
G
T
C
A
G
T
C
T
G
A
C
T
G
A
C
T
G
A
T
C
G
A
C
T
A
G
C
G
A
T
C
A
T
G
A
C
G
T
C
T
A
G
  
Reverse Opposite:  

G
A
T
C
C
G
T
A
G
T
A
C
C
G
T
A
G
A
T
C
A
G
C
T
A
G
C
T
G
A
C
T
G
A
C
T
G
T
C
A
C
G
T
A
C
G
T
A
G
T
A
C
  

|  |  |
| --- | --- |
| p-value: | 1e-47 |
| log p-value: | -1.090e+02 |
| Information Content per bp: | 1.759 |
| Number of Target Sequences with motif | 93.0 |
| Percentage of Target Sequences with motif | 1.95% |
| Number of Background Sequences with motif | 75.0 |
| Percentage of Background Sequences with motif | 0.18% |
| Average Position of motif in Targets | 102.2 +/- 30.8bp |
| Average Position of motif in Background | 97.9 +/- 69.0bp |
| Strand Bias (log2 ratio + to - strand density) | -0.9 |
| Multiplicity (# of sites on avg that occur together) | 1.00 |
| Motif File: | file (matrix) reverse opposite |

### Similar de novo motifs found

|  |  |  |  |  |  |  |  |
| --- | --- | --- | --- | --- | --- | --- | --- |
| Rank | Match Score | Redundant Motif | P-value | log P-value | % of Targets | % of Background | Motif file |
| 1 | 0.916 | G T C A G A T C C T G A A G T C C A G T A C G T A C G T G A C T G T C A C G T A C T G A G A T C T C G A T C G A | 1e-45 | -103.900103 | 1.53% | 0.09% | motif file (matrix) |
| 2 | 0.965 | G A C T A C G T C G A T C G T A C G T A G T C A T C G A C A T G A G C T C T A G A C G T C T A G | 1e-40 | -94.009922 | 1.74% | 0.17% | motif file (matrix) |
| 3 | 0.937 | A T C G A C G T A C G T C A G T G T C A C T G A G T C A T C G A C T A G A C G T C T A G G C A T C A T G A C G T | 1e-35 | -81.499837 | 1.28% | 0.09% | motif file (matrix) |
| 4 | 0.940 | A C G T A C G T A C G T C G T A C G T A C G T A G T C A A C T G A C G T A C T G A C G T | 1e-35 | -80.859046 | 1.64% | 0.19% | motif file (matrix) |
| 5 | 0.875 | C G A T A C G T C G T A C G T A C G T A G T C A A C T G A C G T A C T G A C G T | 1e-28 | -64.588882 | 1.55% | 0.24% | motif file (matrix) |
